# Multi-channel high-order network representation learning research

**DOI:** 10.3389/fnbot.2024.1340462

**Published:** 2024-02-29

**Authors:** Zhonglin Ye, Yanlong Tang, Haixing Zhao, Zhaoyang Wang, Ying Ji

**Affiliations:** School of Computer, Qinghai Normal University, Xining, Qinghai, China

**Keywords:** network representation learning, node embedding, high-order feature, multi-channel learning, graph assimilation

## Abstract

The existing network representation learning algorithms mainly model the relationship between network nodes based on the structural features of the network, or use text features, hierarchical features and other external attributes to realize the network joint representation learning. Capturing global features of the network allows the obtained node vectors to retain more comprehensive feature information during training, thereby enhancing the quality of embeddings. In order to preserve the global structural features of the network in the training results, we employed a multi-channel learning approach to perform high-order feature modeling on the network. We proposed a novel algorithm for multi-channel high-order network representation learning, referred to as the Multi-Channel High-Order Network Representation (MHNR) algorithm. This algorithm initially constructs high-order network features from the original network structure, thereby transforming the single-channel network representation learning process into a multi-channel high-order network representation learning process. Then, for each single-channel network representation learning process, the novel graph assimilation mechanism is introduced in the algorithm, so as to realize the high-order network structure modeling mechanism in the single-channel network representation learning. Finally, the algorithm integrates the multi-channel and single-channel mechanism of high-order network structure joint modeling, realizing the efficient use of network structure features and sufficient modeling. Experimental results show that the node classification performance of the proposed MHNR algorithm reaches a good order on Citeseer, Cora, and DBLP data, and its node classification performance is better than that of the comparison algorithm used in this paper. In addition, when the vector length is optimized, the average classification accuracy of nodes of the proposed algorithm is up to 12.24% higher than that of the DeepWalk algorithm. Therefore, the node classification performance of the proposed algorithm can reach the current optimal order only based on the structural features of the network under the condition of no external feature supplementary modeling.

## 1 Introduction

With the rapid development of the information age, the explosive growth of data has occurred, and the scale of network structure data, as one of the carriers of information, has also increased accordingly. Seeking a convenient method for processing network structure data has become a focal point of investigation. As one of the methods for handling network structure data, network representation learning has attracted widespread attention in recent years. It allows learning a low-dimensional and dense vector for each node in the network, enabling the representation of sparse networks in a concise manner for ease of subsequent task processing.

Early network representation learning research mainly focused on methods based on matrix decomposition (Belkin and Niyogi, 2003). For example, the adjacency matrix or Laplacian matrix of the network is decomposed, and the obtained feature vector is used as the representation of the node (Ng et al., 2002). This type of method can capture the global structure of the network, but has high computational complexity and is not suitable for large-scale networks (Cao et al., 2015a). Therefore, network embedding methods based on random walks have become popular, such as DeepWalk (Perozzi et al., 2014) and Node2Vec (Grover and Leskovec, 2016). These methods generate a sequence of nodes by performing a random walk on the network, and then use word embedding techniques (such as Word2Vec) to encode the nodes into vectors (Mikolov et al., 2013a). This type of method can capture the local structure of the network, has high computational efficiency, and is suitable for large-scale networks (Tang et al., 2015). Subsequently, inspired by work in the field of word representation learning (Mikolov et al., 2010, 2013a,b), Perozzi et al. (2014) introduced deep learning methods into the field of network representation learning, setting off a new upsurge in research in this field.

Benefiting from the rapid development of the field of deep learning, research on network representation learning based on deep learning has also gained increasing attention from researchers. In this process, the concepts of local features and global features gradually surfaced. Local features mainly focus on the relationship between a single node in the network or a small range of nodes, such as the degree of the node, clustering coefficient, etc. The global characteristics start from the perspective of the entire network and consider the overall structure and properties of the network, such as the diameter of the network, average path length, etc. As research progresses, researchers are no longer satisfied with training only on local structural features of the network. Instead, attention has shifted to capturing the global structural features of the network. Currently, due to the relatively insufficient capture of global structural features in existing work, the performance of trained node vectors is limited.

In order to more comprehensively capture the global structural features of the network, we propose a meta-strategy for multi-channel network representation learning, which we call MHNR. Unlike mainstream single-channel learning algorithms, the MHNR algorithm establishes multiple feature channels, one channel models features of one order, and finally fuses features of different orders for learning. Joint modeling learning of local features and high-order features is realized. Specifically, our contributions are as follows.

- Firstly, we reconstruct the original network structure to obtain sub-networks of different layers, providing possibilities for subsequent multi-channel learning.
- Secondly, we perform graph assimilation operations on different sub-layers, enabling us to extract more comprehensive network structural feature information.
- Finally, we propose a meta-strategy learning method that can select appropriate underlying algorithm models for different types of data. We conduct experiments on multiple datasets, verifying the feasibility of this strategy.

## 2 Related works

Early network representation learning algorithms were primarily based on the computation of matrix eigenvectors. However, due to their inherent characteristics, these algorithms faced significant limitations during the computation process, leading to a lack of widespread development in this category. Subsequently, inspired by the field of representation learning, Perozzi et al. (2014) introduced deep learning techniques into network representation learning, proposing the DeepWalk algorithm. The introduction of the DeepWalk algorithm inspired subsequent work, catalyzing the rapid development of the field. In order to alter the random walk pattern of equally probable node selection in the DeepWalk algorithm, Grover and Leskovec (2016) introduced the node2vec algorithm. To address the shortcomings of the DeepWalk algorithm in extracting network features, Tang et al. (2015) proposed the LINE algorithm. This algorithm models the first-order and second-order similarities of the network, comprehensively preserving the global feature information of the network. In order to make the obtained node vectors more suitable for classification tasks, Li et al. (2016) introduced the DDRW algorithm, integrating the DeepWalk algorithm and the maximum-margin classifier. The EPDW algorithm and PDW algorithm proposed by Yin and Yue (2023), respectively improved the DeepWalk algorithm's equally probabilistic way of selecting the next node and the random walk direction. Matrix factorization algorithms play a crucial role in recommendation systems. In the VLDB International Conference of 2011, Professors Jiawei Han from UIUC and Yizhou Sun from UCLA introduced the concept of Heterogeneous Information Network (HIN; Sun et al., 2018). In 2017, Huan Zhao from the Hong Kong University of Science and Technology presented research results on recommendation systems based on the fusion of meta-structures in heterogeneous information networks at KDD (Zhao et al., 2017). During this period, significant progress was made in recommendation algorithms based on matrix fusion (Ma et al., 2008; Zhang and Jiang, 2016; Zhu et al., 2017). Subsequently, some shallow neural network-based network representation learning algorithms were proven to be effective in decomposing the feature matrices of networks. For instance, Levy and Goldberg (2014) and Levy et al. (2015) demonstrated that Word2Vec essentially decomposes the SPPMI matrix. As DeepWalk is an improved version based on Word2Vec, subsequent studies also confirmed that DeepWalk essentially decomposes the network structure feature matrix transformed from the adjacency matrix (Yang and Liu, 2018). Furthermore, based on the insight from DeepWalk's matrix factorization, they introduced a text matrix into the matrix factorization process, enriching the learned network representation with textual feature factors. Building upon the TADW algorithm, MMDW incorporates the maximum-margin theory from classification learning into network representation learning, resulting in vectors that encompass both network structure features and node label information (Tu et al., 2016a). Wang et al. (2017) utilized modular non-negative matrix factorization to integrate community structure and network structure into the representation vectors of networks. Additionally, several algorithms derive network node representations based on matrix factorization, such as SPE (Shaw and Jebara, 2009), HOPE (Ou et al., 2016), GraRep (Cao et al., 2015b), M-NMF (Wang et al., 2017), Deep NMF (Flenner and Hunter, 2018), ULGE (Nie et al., 2017), LLE (Roweis and Saul, 2000), FONPE (Pang et al., 2017), and among others.

In order to further improve the classification performance of network nodes, researchers have turned their attention to the combination of other network information, such as community, higher-order features, text features, etc. Tu et al. (2016b) proposed the CNRL algorithm by capturing the community information hidden in the network to constrain the vector representation of network nodes. The vGraph algorithm proposed by Sun et al. (2019) solves the problem of separating community detection from node representation in current work, so that the trained nodes can obtain better performance. Wang et al. (2021a) proposed the NTF algorithm based on the theory of energy levels, which could better utilize the neighborhood structure characteristics of nodes. The BIGCLAM algorithm proposed by Yang and Leskovec (2013) was able to capture the overlapping communities in the network nodes. Xue et al. (2022) proposed the BiasedWalk algorithm with a preference for random walk, which can make nodes with the same semantics have closer distances in vector space. Zhang et al. (2023) proposed a restartable random walk strategy to enhance the capture of both global and local structural features in networks. Khan et al. (2021) proposed VECODER, a joint learning model based on variational embedding of community discovery and node representation, which utilized community aware node embedding to better detect node communities. Wang et al. (2021b) proposed a framework that could incorporate local structure information into a random walk model, which could effectively reconstruct local subgraphs and capture local structure features.

For other relevant work, please refer to the work of Liu et al. (2022).

## 3 Methodology

### 3.1 DeepWalk

DeepWalk algorithm was inspired by word representation learning and introduced deep learning techniques into the field of network representation learning. It is the most classic and commonly used method in network representation learning, and is also the basis of the MHNR algorithm in the paper. The MRNH algorithm proposed in the paper aims to establish different-order features of the modular network, thereby achieving effective fusion of local features and global features, so that the learned network representation vector contains both low-order and high-order feature factors. Therefore, we will introduce the principle of DeepWalk algorithm here. Of course, the MHNR algorithm can also be improved using other algorithms as a basis.

DeepWalk carries out a random walk on the network structure to obtain a sequence of nodes, and obtains a group of node pairs (*v*_*i*_ | *v*_*k*_) through the sliding window. Skip-Gram algorithm aims to maximize the probability of the occurrence of context nodes when the target nodes are given. Its objective function is as follows:


$$\begin{array}{l} L(V)=\text{arg max}\sum_{i=1}^{N}\sum_{-k\leq \;j\;\leq \;k}\log P(v_{i+j}\;|\;v_{i}),\; \end{array}$$

where, *N* is the total number of nodes in the network, *k* is the size of the sliding window, and *P*(*v*_*i*+*j*_ | *v*_*i*_) is defined by softmax function:


$$\begin{array}{l} P\left(v_{i+j}\;|\;v_{i}\right)=\overset{\;}{\underset{v_{i+j}\in C\left(v_{i}\right)}{\prod }}\frac{\exp\left({\text{v}}_{i+j}^{T}\cdot {\text{v}}_{i}\right)}{\underset{v_{i+j}\in V}{\sum }\exp\left({\text{v}}_{i+j}^{T}\cdot \;{\text{v}}_{i}\right)}, \end{array}$$

where **v**_*i*_ represents the representation vector of node *v*_*i*_, *C*(*vi*) represents the set of context nodes for the target node *v*_*i*_. According to Formula (2), in the process of network training, the whole network node needs to be calculated, which requires a large amount of calculation. Therefore, the negative sampling method is introduced to improve the training efficiency, which is transformed into a binary classification problem. For a set of node pairs, exists:


$$P(v_{i},v_{k})=\left\{ \begin{array}{l} \;P(v_{i},v_{k}),\;\;\;L=1.\\ 1-P(v_{i},v_{k}),\;\;\;L=0. \end{array}\right.$$

*L* = 1 is positive example, it's indicating that the label of node pairs from the same corpus is 1; *L* = 0 is negative example, it's indicating that the label of node pairs from different corpora is 0.

Therefore, the Skip-Gram objective function based on negative sampling is:


$$\begin{array}{l} L(V)=argmax\sum_{i=1}^{N}\sum_{-k\;\leq \;j\;\leq \;k}logP(v_{i+j}|v_{i})\\ {{\;+\sum }^{\text{​}}}_{v_{c}\in Sample}logP(-v_{c}|v_{i}\;), \end{array}$$

where *Sample* represents the set of nodes obtained through negative sampling, and (−*v*_*c*_ | *v*_*i*_) denotes the representation vector of the context node obtained through negative sampling.

### 3.2 High-order network generation

In the current work of network representation learning, the vector representation of nodes is obtained by random walk on the network structure, such as DeepWalk algorithm, etc., which can only obtain the low-order features of the network, thus ignoring the global features of the network.

**Figure 1:**
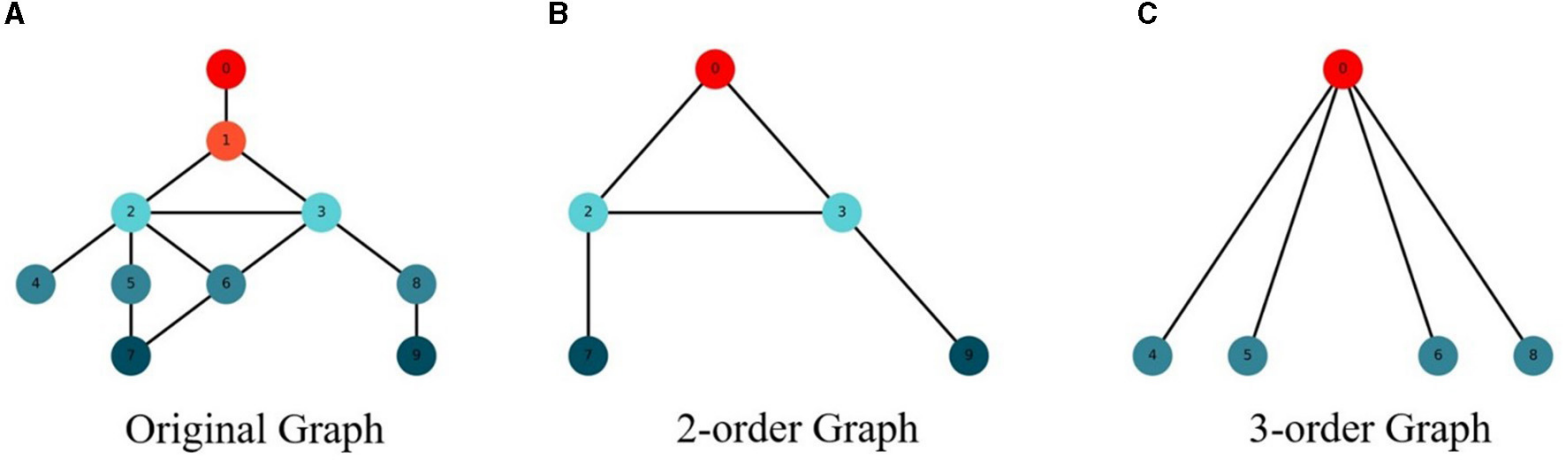
High-order subgraph generation. **(A)** Original graph. **(B)** 2-order graph. **(C)** 3-order graph.

In order to better model the network structure, this paper modeled the M-order structure features of the network on the basis of the low-order network features to obtain the global feature information of the network, so as to improve the performance of the network nodes. The feature extraction diagram of M-order structure of the network is shown in Figure 1.

In order to capture the M-order features of the network, this paper changes the step size of the random walk of the network structure to obtain the structural features of different orders. When the original network is modeled, the step size is set as 1 to obtain the structural characteristics of the original network. When modeling the structural features of the 2-order network, set the step size to 2 to obtain the structural features of the 2-order network. By analogy, the M-order structure feature information of the network can be obtained, so as to model the global structure feature of the network.

### 3.3 MHNR algorithm

**Figure 2:**
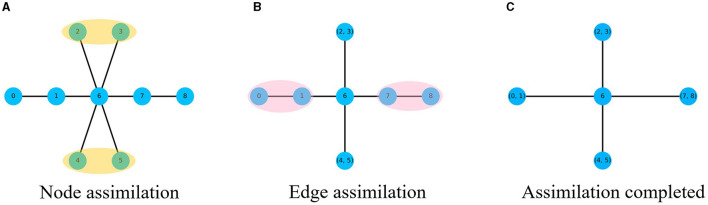
Graph assimilation process. **(A)** Node assimilation. **(B)** Edge assimilation. **(C)** Assimilation completed.

The node sequence obtained by DeepWalk algorithm through random walks in the network is used as the training sample, and the network features collected are local. In order to better integrate global features into the training process, this paper proposes a multi-channel high-order network representation learning algorithm MHNR algorithm. On the basis of the M-order graph generated, MHNR algorithm carries out center point assimilation and edge assimilation operations on the subgraphs of different orders to reduce the huge graph structure into a smaller graph structure. By carrying out random walks on the smaller graph structure, more structural information can be obtained and the quality of network embedding can be optimized. The process of graph assimilation is mainly divided into two types, namely central point assimilation and edge assimilation. The assimilation process is shown in Figure 2.

**Figure 3:**
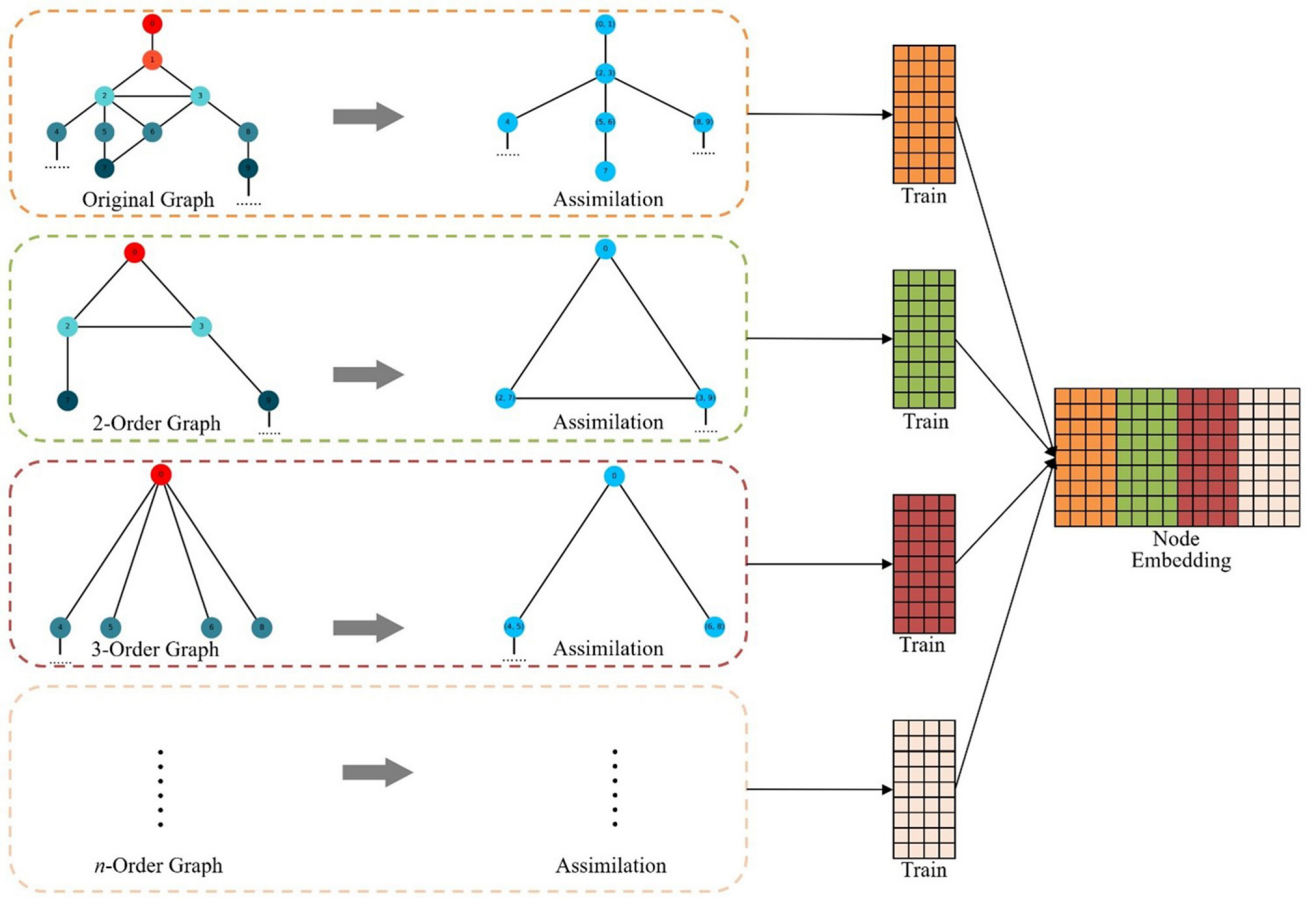
MHNR algorithm.

In order to better coordinate the sequence of assimilation, the MHNR algorithm stipulates that the central point assimilation should be carried out first, and then the side assimilation should be carried out. When the network is not changed, the assimilation will stop. Then a random walk is carried out on the network that completes graph assimilation, and the node sequence of the assimilated nodes is obtained. The obtained node sequence is taken as the input of Skip-Gram, and the corresponding node vector is trained. The framework of MHNR algorithm is shown in Figure 3.

The objective function of the assimilated M-order graph is:


$$\begin{array}{l} L_{m}(V)=argmax\sum_{i=1}^{N}\sum_{-k\;\leq \;j\;\leq \;k}logP(v_{i+j}^{m}|v_{i}^{m})\\ \;{{+\sum }^{\text{​}}}_{v_{c}\in Sample}logP(-v_{c}|v_{i}^{m}\;), \end{array}$$

where, *M* represents the order of the graph used for assimilation. After the training is completed, it is necessary to restore the node vector obtained after the assimilation of different hierarchy graphs. The node vector obtained by training has two forms, one is the node vector representation without assimilation, and the other is the vector representation after assimilation. For the vector representation of node vector after assimilation, the node vector obtained from training can be directly used to represent the node vector before assimilation. The node vector of each order is obtained by training, and the final node vector of MHNR is obtained by splicing the node vector obtained by training of each order. The vector representation of network nodes is obtained by concatenating node vectors of all orders:


$$\begin{array}{l} \text{E}={\text{E}}_{0}⊕{\text{E}}_{2}⊕\cdots ⊕{\text{E}}_{\text{i}}⊕\cdots ⊕\;{\text{E}}_{\text{n}}, \end{array}$$

where *E* represents the final node vector representation for MHNR. The pseudocode can be found in Algorithm 1.

**Algorithm 1:**
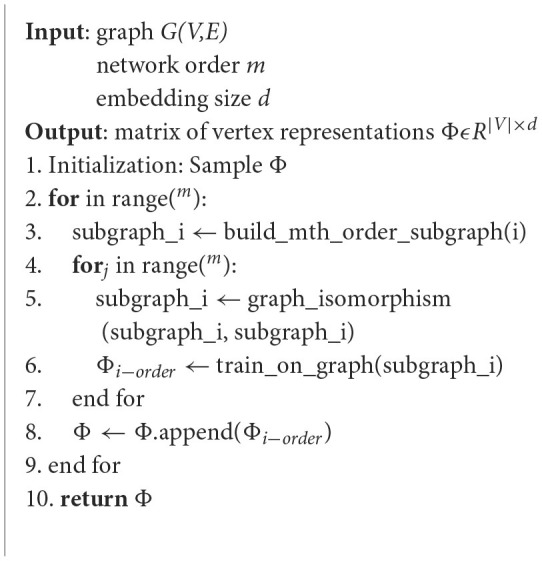
MHNR.

### 3.4 Complexity analysis

A distinctive feature of MHNR is its adaptive time complexity, which is contingent upon the specific underlying model chosen for the task. In this context, we opt to illustrate the time complexity analysis of MHNR algorithm by taking the DeepWalk algorithm as a representative example.

DeepWalk algorithm's time complexity calculation can be divided into two parts. The first part involves random walk modeling, where the algorithm performs T steps of random walks for each node V in the network, resulting in a time complexity of *O*(V^*^T). The second part pertains to model training, with a time complexity of *O*(V^*^E), where V is the number of nodes, E is the average number of neighbors, and it is typically proportional to the size of the input data. Therefore, the time complexity of the DeepWalk algorithm is expressed as *O*(V^*^T) + *O*(V^*^E). Consequently, the time complexity of the MHNR algorithm based on the DeepWalk model is *O*(V^*^T) +Õ(V^*^E).

## 4 Experiments and results

### 4.1 Datasets

In order to verify the feasibility of the proposed algorithm, experiments were performed on Citeseer, Cora, and DBLP (V4) data sets. The selected data set is the real network data set, and the relevant indicators of each data set are shown in Table 1.

**Table 1:** Data description.

**Ds**.	**Nodes number**	**Edges number**	**Average degree**	**Network diameter**	**Average aggregation coefficient**
Citeseer	3,312	4,732	2.857	28	0.257
Cora	2,708	5,429	4.01	19	0.293
DBLP	3,119	39,516	25.339	14	0.259

If there're have isolated nodes in the network, the random walk results of MHNR algorithm and comparison algorithm will be affected. In order to ensure the accuracy of the experimental results, the isolated nodes in the DBLP data set were deleted. As can be seen from Table 1, Citeseer data set, Cora data set, and DBLP data set have similar number of nodes, but there are significant differences in the number of edges in the network. Citeseer data sets and Cora data sets are sparse network data sets, while DBLP data sets are dense network data sets. Therefore, the three selected data sets can simulate the experimental effects of the algorithm under different conditions.

### 4.2 Contrast algorithm

The MHNR algorithm proposed in this paper is a network representation learning algorithm based on network structure, which does not use network node tags, text content, known community tags and other information. Therefore, the comparison algorithm used in this section is mainly a network representation learning algorithm based on structure mining. The comparison algorithms are introduced as follows.

- **DeepWalk (Perozzi et al.**, **2014****):** DeepWalk algorithm introduced the technology of deep learning into network representation learning for the first time. This algorithm proved that the node sequence obtained by random walk on the network followed the exponential law, just like the words in the text. Then the node sequence was put into the neural network to obtain the node vector representation.
- **LINE (Tang et al.**, **2015****):** DeepWalk algorithm for random walks on the network is sparse. In order to solve this problem, the 2-order similarity is introduced into LINE algorithm. The 2-order similarity defines that the more common neighbors between two nodes, the closer they should be in vector space.
- **node2vec (Grover and Leskovec**, **2016****):** node2vec algorithm improves the random walk mode of DeepWalk algorithm on the basis of DeepWalk algorithm. Two random walk strategies are proposed. Two hyperparameters and are introduced to control the proportion of breadth-first strategy and depth-first strategy, respectively.
- **GraRep (Sun et al.**, **2018****):** In order to better capture K-order structure information of the network, GraRep algorithm adopts the method of matrix decomposition to embed nodes. This algorithm can deal with weighted networks, and at the same time, this algorithm can integrate the global structure information of the network during the training process.
- **DeepWalk+NEU (Yang et al.**, **2017****):** This algorithm is the combination of DeepWalk algorithm and NEU algorithm. In this comparison algorithm, DeepWalk algorithm is first used to train the network to obtain the vector representation of the network nodes, and then NEU algorithm is used to carry out the high-order transformation of the obtained network embedding.
- **EPDW (Yin and Yue**, **2023****):** The EPDW algorithm improves the wandering mode of DeepWalk algorithm to select the next hop node with equal probability, and introduces the gambling wheel method to change the probability of selecting the next hop node. This method can select the next hop node more reasonably.
- **PDW (Yin and Yue**, **2023****):** The PDW algorithm changes the undirected network into a directed network, and introduces multiple hyperparameters to control the direction of the random walk. The hyperparameters are determined by the edge weights of the central node and its neighbors, and attenuates the weight of the edge traveled while restraining the probability of returning to the previous hop node.
- **MHNR**_**2**_ **(DeepWalk):** Algorithm of this paper, the original network structure channel and the 2-order high-order network structure channel are simultaneously modeled. MHNR algorithm uses DeepWalk as the meta-algorithm when modeling the relationship between network nodes.
- **MHNR**_**3**_ **(DeepWalk):** Algorithm of this paper, the original network structure channel and 2-order and 3-order high-order network structure channel are simultaneously modeled. MHNR algorithm uses DeepWalk as the meta-algorithm when modeling the relationship between network nodes.
- **MHNR**_**5**_ **(DeepWalk):** Algorithm of this paper, the original network structure channel and 2-order, 3-order, 4-order, and 5-order high-order network structure channel are simultaneously modeled. MHNR algorithm uses DeepWalk as the meta-algorithm when modeling the relationship between network nodes.

### 4.3 Experimental parameter setting

In order to verify the generalization ability of MHNR algorithm, experimental verification was carried out on Citeseer, Cora, and DBLP data sets. The data sets were divided into nine proportional training sets with a ratio from 10 to 90% and an interval of 10%. The remaining data were used as test sets. Moreover, SVM provided by Fan et al. (2008) in LIBLINEAR 22 was selected as the classifier to conduct a multi-vertex classification accuracy experiment on network embedding results. The algorithm was set to walk 40 nodes each time as the length of random walk sequence, the number of random walks was set to 10, the size of sliding window was set to 5, the number of negative samples was set to 5, and the minimum node frequency was set to 5. In the PDW algorithm, three groups of hyperparameter control PDW algorithm are set, in which the inhibition coefficient of the previous hop node in the random walk return walk sequence is set as 5, 10, 20, and the walk probability attenuation coefficient is set as 0.05, 0.05, and 0.1. In EPDW algorithm, the random walk length is set to 40, 60, and 80, respectively. At the same time, the dimension of node vector in each channel of MHNR algorithm is set to 100 dimensions. In order to ensure the accuracy of the experimental results, the experiment was repeated for 10 times, and the average value of the results of 10 times was taken as the final result of the experiment.

### 4.4 Experimental results and analysis

In the experiment to verify the results of MHNR algorithm, the three selected evaluation data sets are real network data sets. Tables 2–4, respectively show the experimental results of MHNR algorithm and comparison algorithm in different data sets and different proportion test sets.

**Table 2:** Citeseer data set experimental results (%).

**Algorithm**	**10%**	**20%**	**30%**	**40%**	**50%**	**60%**	**70%**	**80%**	**90%**	**Avg**.
DeepWalk	47.55	50.24	51.89	52.32	53.66	53.16	53.75	53.91	54.62	52.34
LINE	41.22	44.61	47.88	49.15	52.18	53.49	53.87	53.25	53.91	49.51
DeepWalk+NEU	48.49	51.23	52.48	53.90	53.54	54.65	54.57	54.43	55.86	53.24
GraRep (*K* = 3)	45.07	50.95	53.40	54.21	54.87	55.75	55.54	55.15	54.22	53.24
node2vec	50.83	52.58	54.28	54.45	55.71	56.18	55.58	56.22	56.62	54.72
EPDW (*l* = 40)	51.92	53.84	55.16	55.81	56.86	57.09	57.52	57.90	58.33	56.05
EPDW (*l* = 60)	51.26	53.40	54.93	55.76	56.59	57.09	57.06	57.22	58.56	55.76
EPDW (*l* = 80)	50.53	53.57	54.64	55.08	55.66	56.20	56.34	57.15	57.33	55.16
PDW (*p* = 5, *q* = 0.05)	53.16	55.19	55.74	56.33	57.26	57.93	57.98	57.96	57.65	56.58
PDW (*p* = 10, *q* = 0.05)	52.89	54.99	55.70	56.66	56.26	56.90	57.37	57.42	57.65	56.20
PDW (*p* = 20, *q* = 0.1)	53.65	54.77	55.41	55.87	56.07	56.98	56.74	57.89	57.22	56.07
MHNR_1_ (DeepWalk)	54.43	56.19	57.53	59.03	59.08	59.65	59.34	59.21	60.69	58.35
MHNR_3_ (DeepWalk)	55.89	58.92	59.38	60.52	60.91	62.31	62.53	62.58	62.18	60.58
MHNR_5_ (DeepWalk)	**56.10**	**59.48**	**61.55**	**61.72**	**62.42**	**63.00**	**62.90**	**64.17**	**65.80**	**61.90**

Bold values represent the highest value in the column.

**Table 3:** Cora data set experimental results (%).

**Algorithm**	**10%**	**20%**	**30%**	**40%**	**50%**	**60%**	**70%**	**80%**	**90%**	**Avg**.
DeepWalk	67.60	72.09	74.47	75.07	76.68	76.74	77.44	78.08	77.70	75.10
LINE	64.25	68.38	70.11	71.34	73.26	75.81	75.62	77.73	79.51	68.75
DeepWalk+NEU	69.29	74.74	76.08	77.34	77.76	78.59	78.83	79.37	79.07	76.79
GraRep (*K* = 3)	72.60	77.34	78.34	79.39	79.43	80.30	80.32	80.68	79.88	78.70
node2vec	69.31	73.24	74.13	75.60	76.13	76.58	76.45	77.45	77.44	75.15
EPDW (*l* = 40)	72.50	75.86	76.84	77.64	77.98	78.65	78.94	79.23	79.65	77.48
EPDW (*l* = 60)	72.35	75.40	76.46	77.52	77.42	77.96	77.96	78.13	78.78	76.89
EPDW (*l* = 80)	71.85	74.89	76.07	76.72	77.08	77.27	78.01	78.43	78.25	76.51
PDW (*p* = 5, *q* = 0.05)	75.66	78.11	79.40	80.19	80.52	80.97	80.58	80.91	81.00	79.70
PDW (*p* = 10, *q* = 0.05)	75.96	78.79	79.51	79.99	80.32	80.42	81.81	81.57	81.63	80.00
PDW (*p* = 20, *q* = 0.1)	76.50	78.56	79.86	79.91	80.20	80.78	81.40	81.74	81.07	80.00
MHNR_1_ (DeepWalk)	75.86	78.81	79.55	80.57	80.62	81.34	81.02	81.92	81.81	80.17
MHNR_3_ (DeepWalk)	**76.56**	**78.98**	**80.08**	80.49	**81.62**	**81.77**	**82.50**	82.55	**82.81**	**80.82**
MHNR_5_ (DeepWalk)	75.94	78.58	79.83	**81.02**	81.39	81.63	82.07	**82.64**	82.41	80.61

Bold values represent the highest value in the column.

**Table 4:** DBLP data set experimental results (%).

**Algorithm**	**10%**	**20%**	**30%**	**40%**	**50%**	**60%**	**70%**	**80%**	**90%**	**Avg**.
DeepWalk	76.73	79.47	80.78	81.24	82.11	81.60	82.60	83.24	82.60	81.15
LINE	73.28	75.15	76.93	77.42	78.06	78.65	78.92	80.13	80.52	77.67
DeepWalk+NEU	80.86	81.64	82.10	83.84	83.89	83.79	83.89	84.45	84.02	83.16
GraRep (*K* = 3)	81.59	83.13	84.33	84.07	84.03	84.42	85.24	85.51	85.05	84.15
node2vec	83.17	83.10	83.32	83.62	84.84	84.91	84.29	84.82	84.82	84.10
EPDW (*l* = 40)	84.06	84.51	84.82	85.31	85.04	85.32	85.60	85.91	86.20	85.20
EPDW (*l* = 60)	**84.50**	**85.32**	**85.74**	**85.99**	**86.12**	**86.09**	85.91	86.18	86.44	**85.81**
EPDW (*l* = 80)	84.05	85.04	85.44	85.66	85.69	85.97	**86.16**	**86.55**	**86.54**	85.68
PDW (*p* = 5, *q* = 0.05)	82.91	83.29	83.79	83.86	84.28	84.07	84.96	84.41	84.57	84.02
PDW (*p* = 10, *q* = 0.05)	82.58	83.26	83.83	84.19	84.16	84.47	84.36	85.30	85.34	84.17
PDW (*p* = 20, *q* = 0.1)	82.74	83.45	84.01	84.77	84.57	84.23	84.09	85.85	85.08	84.31
MHNR_1_ (DeepWalk)	82.44	82.92	83.28	83.52	83.88	84.23	84.03	84.56	86.14	83.89
MHNR_3_ (DeepWalk)	82.38	83.54	84.19	84.51	84.78	85.12	85.75	85.30	85.95	84.61
MHNR_5_ (DeepWalk)	82.28	84.00	84.47	84.95	85.35	85.08	85.07	85.91	85.89	84.78

Bold values represent the highest value in the column.

From the experimental results on Citeseer data set, it can be found that MHNR algorithm has the best node classification performance when trained in the 5-order multi-channel network. Compared with DeepWalk algorithm, MHNR algorithm improved by 9.56%. Compared with EPDW algorithm, it improves by 6.14%. Compared with PDW algorithm, the improvement is 5.32%. From the experimental results on Cora data set, it can be found that MHNR algorithm has the best node classification performance in 3-order multi-channel network training. Compared with DeepWalk algorithm, MHNR algorithm improved by 5.72%. Compared with EPDW algorithm, it improves by 3.34%. Compared with PDW algorithm, 0.82% improvement. From the experimental results on DBLP data set, it can be found that MHNR algorithm has the best node classification performance in the 5th order multi-channel network training. Compared with DeepWalk algorithm, MHNR algorithm improved by 3.36%. But it is 1.03% worse than EPDW algorithm. Compared with PDW algorithm, it is improved by 0.47%.

From the experimental results, it can be observed that the proposed MHNR algorithm performs well overall on large-scale sparse datasets. This is because in large-scale sparse datasets, the connections between nodes are not tightly knit, and there may be longer paths between two nodes in the network. This makes it challenging to capture the relationship between two nodes. However, the MHNR algorithm can reconstruct the original network, thereby capturing more comprehensive features of the network and discovering relationships between nodes more effectively. Therefore, the experimental results of the MHNR algorithm are better on large-scale sparse datasets. Additionally, due to the denser relationships between two nodes in dense datasets, the experimental results of MHNR on dense datasets are slightly worse compared to other algorithms. The experimental results clearly indicate that the MHNR algorithm is more suitable for handling large-scale sparse datasets.

The experiment achieves the expected experimental results, indicating that the MHNR algorithm based on multi-channel high order network can retain the characteristic information of the network to a great extent, and the trained nodes are more suitable for the work of subsequent tasks.

**Figure 4:**
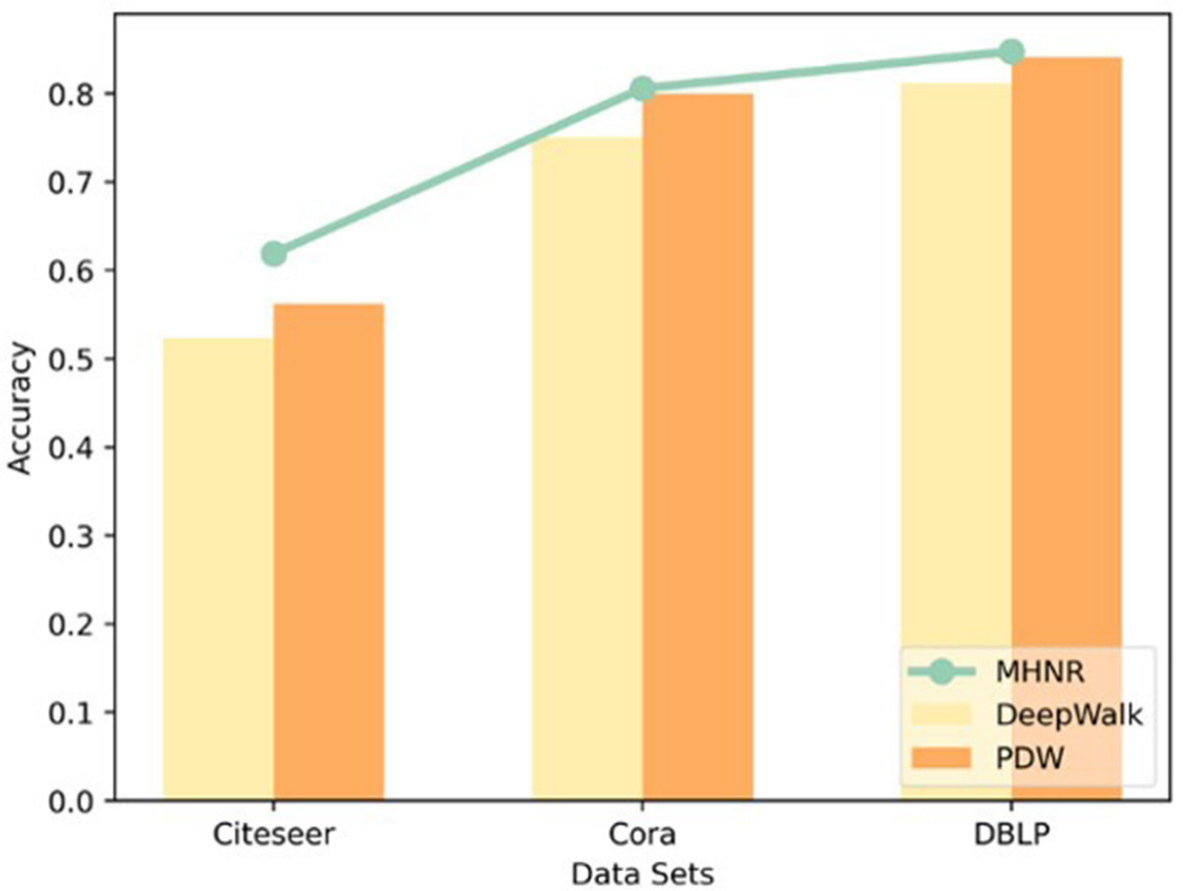
Experimental result.

As can be seen from Figure 4, the algorithm proposed in this paper has obvious advantages in experiments on different data sets. The main reason is that the algorithm in this paper adopts the multi-channel mechanism to model the high-order relationship between network nodes, and adopts the graph assimilation mechanism to model the high-order relationship between network nodes on a single-channel again, which can retain the characteristic information of the network to a great extent. The trained node vector has better classification performance. Therefore, the algorithm in this paper shows very good node classification performance

Node2vec, PDW, and EPDW all improve the machine learning performance of the DeepWalk algorithm by improving the random walk process. However, these two algorithms mainly capture neighboring nodes with closer relationships between nodes, and their essence is still a type of low order network representation learning algorithm. The MHNR algorithm proposed in this paper is a network representation learning algorithm that can train both low-order and high-order features simultaneously, so machine learning can outperform existing node2vec, PDW, and EPDW algorithms.

### 4.5 Visualization analysis

The visual analysis of the training results of the algorithm can observe the classification effect of the algorithm more directly. In this section, visualization analysis experiments were performed on Citeseer, Cora and DBLP data sets, and algorithms such as DeepWalk, EPDW and PDW were selected as comparison algorithms. In MHNR algorithm, 5-channel were selected to train data sets. DeepWalk and node2vec algorithms are selected as the basic algorithm models of MHNR algorithm.

**Figure 5:**
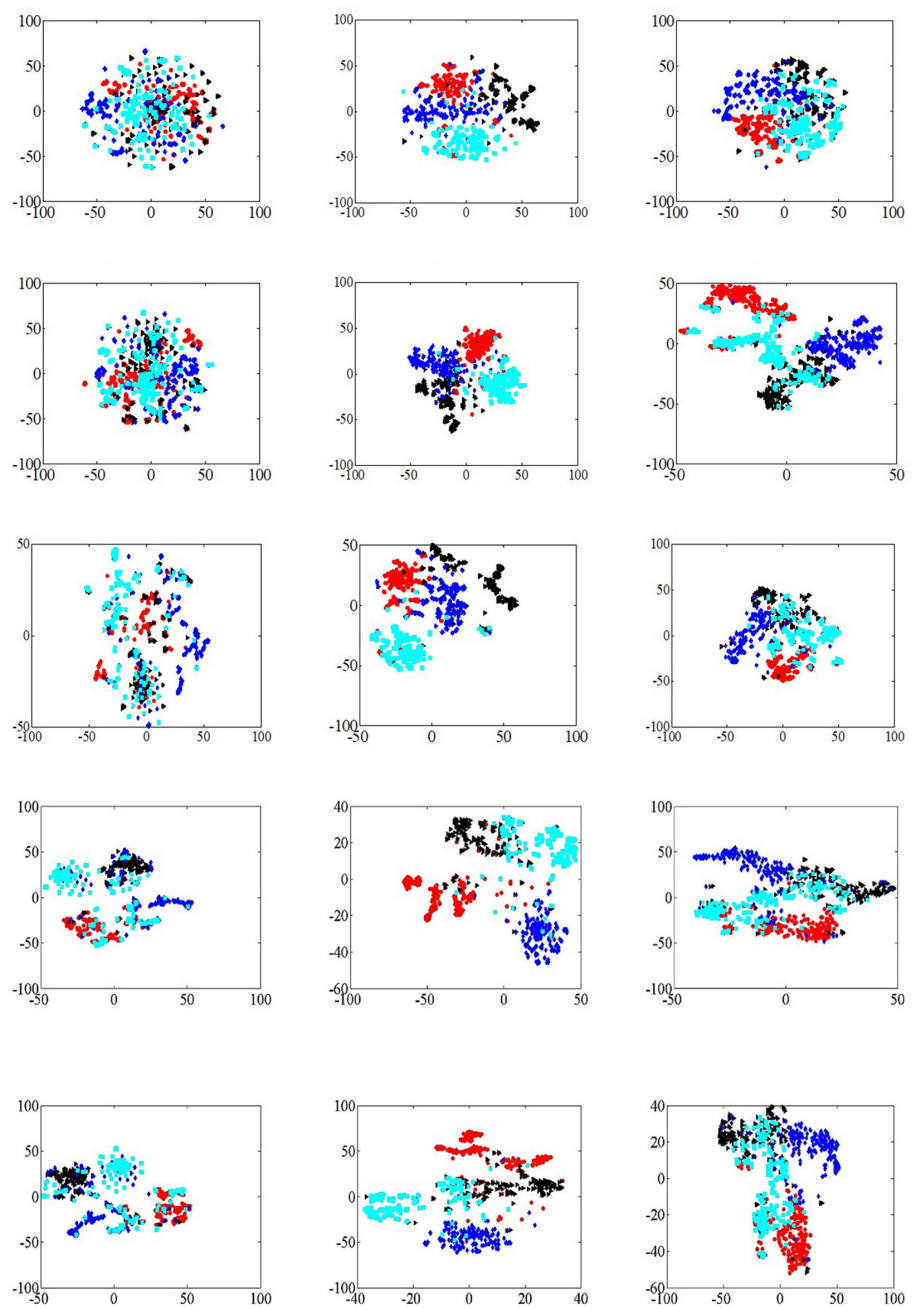
Network visualization. **(A)** DeepWalk (Citeseer). **(B)** DeepWalk (Cora). **(C)** DeepWalk (DBLP). **(D)** EPDW (Citeseer). **(E)** EPDW (Cora). **(F)** EPDW (DBLP). **(G)** PDW (Citeseer). **(H)** PDW (Cora). **(I)** PDW (DBLP). **(J)** MHNR (Citeseer). **(K)** MHNR (Cora; DeepWalk). **(L)** MHNR (DBLP). **(M)** MHNR (Citeseer). **(N)** MHNR (Cora; node2vec). **(O)** MHNR (DBLP).

In the analysis of visualization tasks, four categories are randomly selected in three data sets, and 200 nodes are randomly selected in each category. Meanwhile, t-SNE (Der Maaten and Hinton, 2008) is selected as the node dimension reduction algorithm of visualization tasks. The experimental results of visualization analysis are shown in Figure 5.

The DeepWalk algorithm was able to cluster nodes of the same class in the visualization results of Cora and DBLP data sets, but the visualization results of Citeseer data sets were poor. The visualization results of EPDW algorithm on Citeseer data sets were similar to those of DeepWalk algorithm, but on Cora and DBLP data sets, EPDW algorithm could well gather nodes of the same category together, and there were obvious clustering boundaries. In the visualization result of PDW algorithm on Citeseer data set, there is a significant distance between nodes of the same category and other nodes of the same category, but it fails to gather nodes of the same category well. On Cora and DBLP data sets, PDW algorithm can gather nodes of the same category together, but on Cora data set, The node clustering boundary trained by PDW algorithm is not clear. MHNR algorithms based on different underlying frameworks can cluster nodes of the same category together with obvious clustering boundaries in the visualization results of Citeseer, Cora and DBLP data sets, which further verifies the feasibility of MHNR algorithm.

The MHNR algorithm can model features of different orders, which include both low-order and high-order features. Therefore, the MHNR algorithm can perceive the relationships between farther nodes. However, the DeepWalk algorithm only models the relationship between first-order neighbor nodes and second-order neighbor nodes, so it is a low-cost feature modeling algorithm. The MHNR algorithm can model high-order features, enabling it to capture community information between nodes. Therefore, in visualization tasks, the MHNR algorithm can perform quite well.

### 4.6 Vector length sensitivity analysis

**Figure 6:**
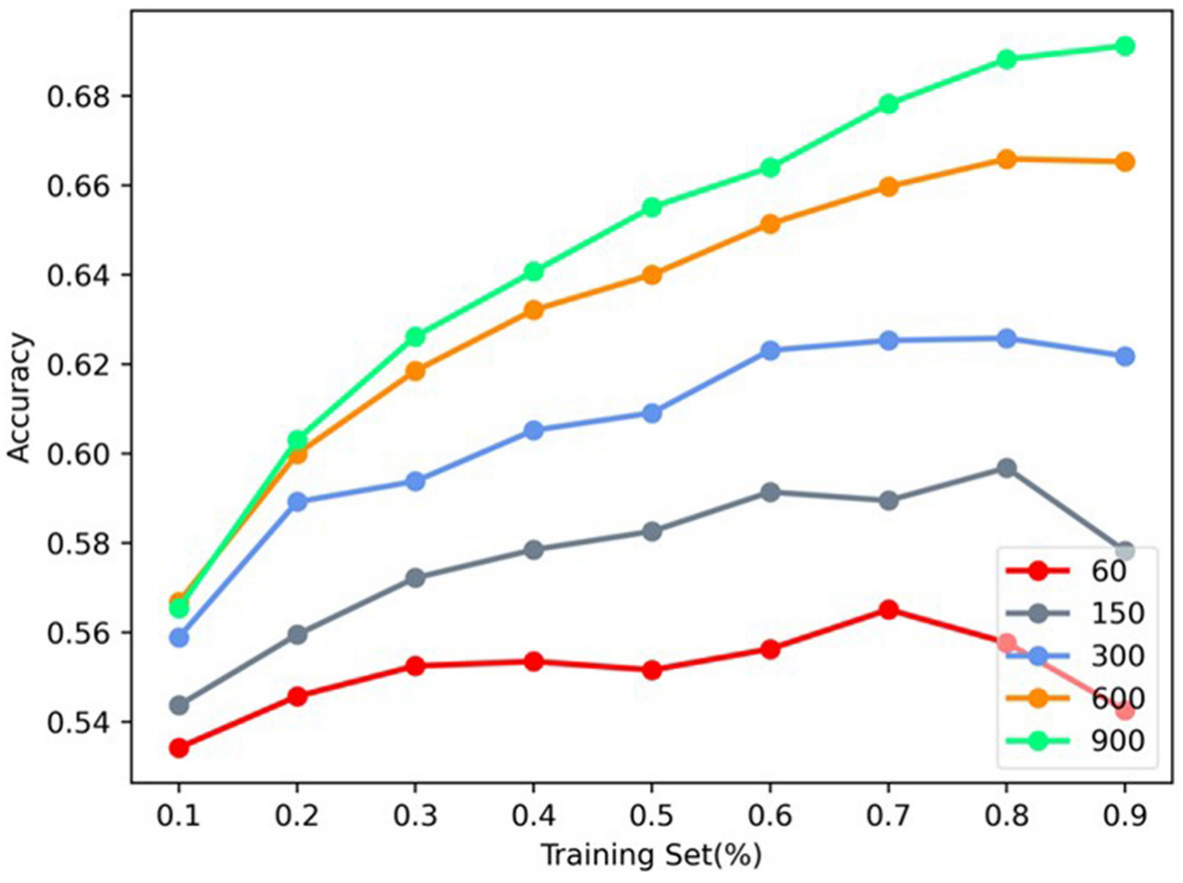
Vector length sensitivity analysis.

In this section, the influence of vector dimension of MHNR algorithm on model accuracy at Citeseer was analyzed. The network structure characteristics of 3-channels modeled by MHNR algorithm were set. The vector dimension of single-channel was set as 20, 50, 100, 200, and 300, and the final vector dimension was 60, 150, 300, 600, and 900, respectively. The specific experimental results are shown in Figure 6.

We found that, as the length of network representation vector increased, the node classification performance of MHNR algorithm became higher and higher. In the node classification experiment, the length of the representation vector of MHNR algorithm was set to 300, and in this experiment, we set the size of the network node representation vector to different values. We found that, when the representation vector size of MHNR algorithm is 60, its node classification performance is the worst, and when the representation vector size of MHNR algorithm is 900, its node classification performance is the best.

### 4.7 Sensitivity comparison of base model

The MHNR algorithm in this paper is trained based on node2vec algorithm and DeepWalk algorithm, respectively. In other words, node2vec algorithm and DeepWalk algorithm are, respectively used to model the relationship between nodes in each single-channel, and then the node classification performance obtained by different meta-algorithms is compared.

**Figure 7:**
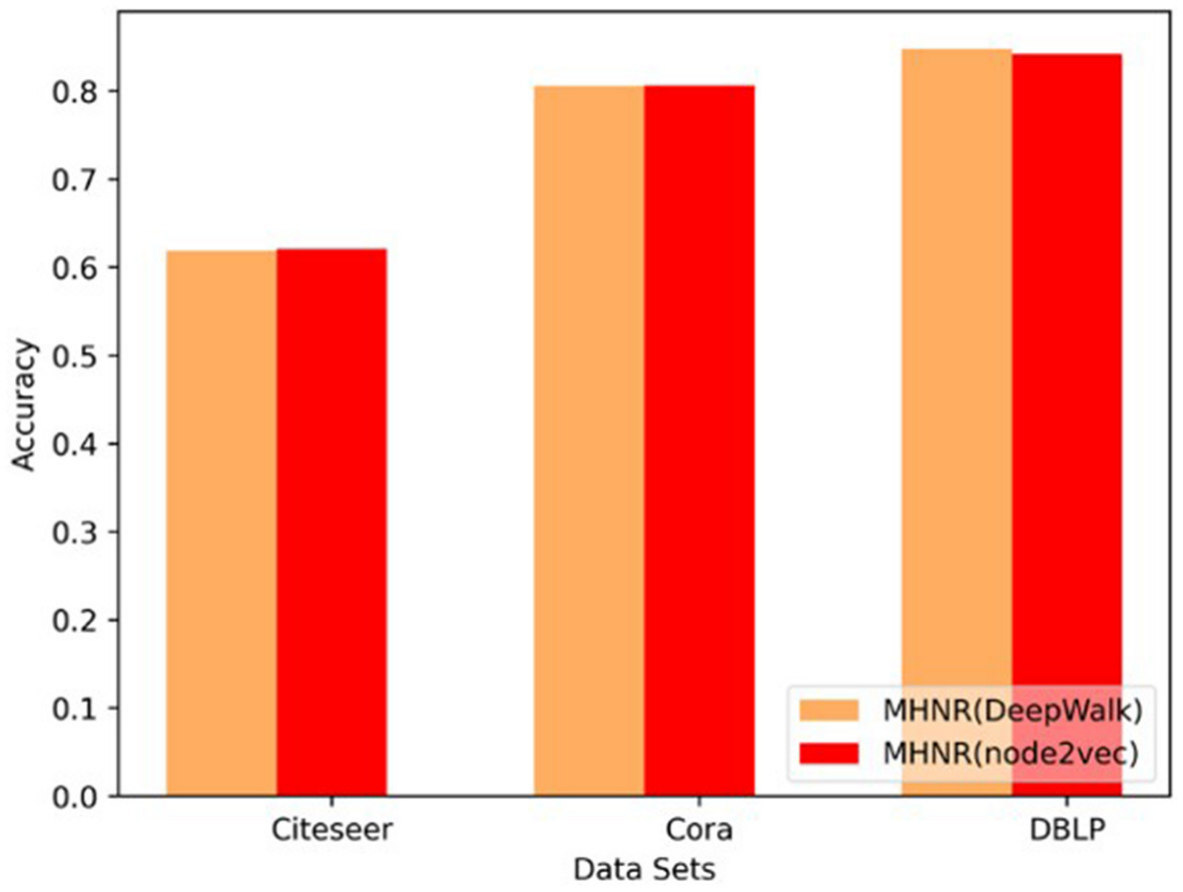
Sensitivity comparison of base model.

The only difference between DeepWalk and node2vec is that the random walk process is different, and the underlying models are the same. In this paper, Skip-Gram and Negative Sampling are adopted to implement the DeepWalk and node2vec algorithms. It can be seen from Figure 7 that, on Citeseer and Cora data sets, MHNR algorithms based on different stratigraphic frameworks show little difference in node classification performance obtained by training on different proportion training sets. However, experimental results on dense data set DBLP show that, the experimental results of the DeepWalk stratigraphic framework algorithm have obtained better node classification performance.

## 5 Conclusion

Based on the original network structure, this paper proposes a multi-channel high-order network representation learning algorithm MHNR. The algorithm takes the lead in capturing the high-order structural features of the network, and at the same time carries out the graph assimilation of the high-order structural features of different orders, respectively, and models the network after the graph assimilation of different orders to obtain the structural features of different orders. The final vector representation of the nodes in the network is composed of node vectors of different orders. The MHNR algorithm is verified by experiments on three real network data sets, and the experimental results are 61.9, 80.61, and 84.78%, respectively, and the expected experimental results are obtained. In the visualization task, MHNR algorithm has good node classification performance, which further proves the feasibility of the algorithm. However, the algorithm still has some limitations, such as:

(1) Dependency on network structure: The MHNR algorithm primarily relies on the network structure for node representation learning, without leveraging additional information like node labels or text content. This may result in suboptimal performance in scenarios lacking sufficient structural information or with a strong dependence on node content, compared to algorithms that consider a variety of information.
(2) Channel selection issue: In the MHNR algorithm, choosing an appropriate number of channels for training is crucial, and the selection may impact the algorithm's performance.

Future directions for improvement include:

(1) Integration of multimodal information: Consider integrating multimodal information, such as node labels and text content, into the MHNR algorithm to enhance its adaptability and generalization. This could potentially improve the algorithm's performance in a broader range of application scenarios.
(2) Adaptive channel selection: Introduce an adaptive mechanism for channel selection, determining the appropriate number of channels based on dataset characteristics or through automatic adjustments to enhance the algorithm's robustness.

## Data availability statement

The original contributions presented in the study are included in the article/supplementary material, further inquiries can be directed to the corresponding author.

## Author contributions

ZY: Conceptualization, Methodology, Software, Writing – review & editing. YT: Conceptualization, Data curation, Writing – original draft. HZ: Writing – review & editing. ZW: Data curation, Investigation, Writing – review & editing. YJ: Data curation, Investigation, Writing – review & editing.
